# Overexpression of PD‐L1 causes germ cells to slough from mouse seminiferous tubules via the PD‐L1/PD‐L1 interaction

**DOI:** 10.1111/jcmm.17305

**Published:** 2022-04-05

**Authors:** Lian Fang, Rui Feng, Weiye Liang, Fang‐Fang Liu, Gan‐lan Bian, Caiyong Yu, Hongmin Guo, Yihui Cao, Mingkai Liu, Jia Zuo, Yinglong Peng, Jie Zhao, Rui‐Xia Sun, Jiajie Shan, Jian Wang

**Affiliations:** ^1^ Department of Neurobiology School of Medicine South China University of Technology Guangzhou China; ^2^ School of Biomedical Sciences LKS faculty of Medicine The University of Hong Kong Pokfulam Hong Kong SAR; ^3^ Institue of Neurosciences The Fourth Military Medical University Xi’an China; ^4^ 26487 Institute of Medical Research Northwestern Polytechnical University Xi'an China; ^5^ 12644 Military Medical Innovation Center Fourth Military Medical University Xi'an China; ^6^ Department of Reproductive Medicine Liaocheng People’s Hospital Liaocheng China; ^7^ School of Biomedical Sciences and Engineering South China University of Technology Guangzhou China; ^8^ Bioscience Laboratory BIOS bioscience and Technology Limited Company Guangzhou China

**Keywords:** adhesion, germ cells, PD‐L1, PD‐L1/PD‐L1, transgenic

## Abstract

Spermatogenesis is a cyclical process in which different generations of spermatids undergo a series of developmental steps at a fixed time and finally produce spermatids. Here, we report that overexpression of PD‐L1 (B7 homolog1) in the testis causes sperm developmental disorders and infertility in male mice, with severe malformation and sloughing during spermatid development, characterized by disorganized and collapsed seminiferous epithelium structure. PD‐L1 needs to be simultaneously expressed on Sertoli cells and spermatogonia to cause spermatogenesis failure. After that, we excluded the influence of factors such as the PD‐L1 receptor and humoral regulation, confirming that PD‐L1 has an intrinsic function to interact with PD‐L1. Studies have shown that PD‐L1 not only serves as a ligand but also plays a receptor‐like role in signal transduction. PD‐L1 interacts with PD‐L1 to affect the adhesive function of germ cells, causing malformation and spermatid sloughing. Taken together, these results indicate that PD‐L1 can interact with PD‐L1 to cause germ cell detachment and male infertility.

## 1 INTRODUCTION

Spermatogenesis is a complex process.^1^, ^2^ During spermiogenesis, haploid round spermatids undergo an elongation phase and are converted into mature sperm.^3^ The mouse is an excellent model organism for studying human genes because the vast majority of genes and processes involved in sperm production appear to be conserved between mice and men.^4^ There are certain advantages to studying spermatogenesis and infertility in transgenic mice.^5^, ^6^ Nonetheless, spermatogenesis, the process of spermatid development, remains incompletely understood.

In mice and rats, spermatids undergo 2–3 weeks of cellular differentiation to form mature elongated spermatids, which are eventually released from the seminiferous epithelium through a process called spermiation.^7^, ^8^, ^9^ Spermatids are interconnected with Sertoli cells during the development by intercellular bridges.^10^ These cell junctions include occluding junctions, adhering junctions and communicating junctions.^11^, ^12^ Among them, ectoplasmic specialization (ES) and the tubulobulbar complex (TBC) are the two most studied types of adherens junctions in the testis. In particular, at stage VIII of spermiogenesis in rats and mice, spermiogenesis is interconnected with ES to facilitate head development and sperm cell motility.^13^

PD‐L1 (programmed cell death ligand 1, B7‐H1) was first discovered in 1997 and is widely expressed as a transmembrane protein.^14^ Subsequent studies revealed that PD‐L1 plays a key role in regulating the immune system.^15^ PD‐L1 interacts with the corresponding receptor PD‐1 (Programmed cell death protein 1) expressed on the surface of activated T cells and B lymphocytes, which delivers inhibitory signals to mediate the immune escape of tumour cells and resistance to conventional chemoradiotherapy.^16^ B7‐1, a member of the B7 family, is proposed as another molecule that can bind with PD‐L1 via cis‐interaction and mediate the function of the immune system.^17^ PD‐L1 is mostly deemed to mediate PD‐1 and CD80 to transduce signals. Tumour cells with PD‐L1 can still resist the attack of T lymphocytes, even in the absence of PD‐1.^18^ Moreover, it has been reported that the intracellular segment of PD‐L1 is the signal transduction domain, which can promote proliferation and resist the proapoptotic effects of interferons, further supporting the role of PD‐L1 in signal transduction.^19^, ^20^ The effect is also reflected in the involvement of PD‐L1 in regulating the growth, proliferation, migration and invasion of tumour cells via the EMT, PI3K/Akt/mTOR and Ras/Erk signalling pathways.^21^, ^22^ With further research on PD‐L1, researchers have found that PD‐L1 has a certain influence on reproduction, but research has mostly focused on maintaining maternal‐foetal tolerance during early pregnancy and preventing pregnancy complications such as pregnancy‐induced hypertension syndrome.^23^, ^24^

The spermatogenesis process is regulated by multiple hormones and local factors as well as by direct interactions between spermatogenic and Sertoli cells.^25^, ^26^ Interference with cell–cell interactions in the testis can affect germ cell movement within the epithelium. If the spermatid is prematurely induced to release into the tubule lumen, the zygote will fail to form. On the contrary, if germ cells are forced to attach to the seminiferous epithelium for longer than it takes to complete their development, they degenerate and are eventually phagocytosed by Sertoli cells. We found that in the testis, overexpression of PD‐L1 in the seminiferous and Sertoli cells of the seminiferous tubules caused spermiation failure and male infertility, whereas overexpression of PD‐L1 only in the spermatogonia did not cause the above phenomenon. These findings led to the hypothesis that PD‐L1 may bind to PD‐L1 on Sertoli cells in a self‐interacting way during the early stages of spermatogenesis and thereby cause sperm cell sloughing. Their significance is also relevant for germ cell movement through Sertoli–Sertoli and Sertoli‐germ cell interactions in the seminiferous epithelium at different stages of spermatogenesis.^27^

We studied PD‐L1 transgenic mice to dissect the role of PD‐L1 in spermatogenesis and found that PD‐L1 can interact with PD‐L1. We found that the overexpression of PD‐L1 can cause complete infertility in male mice. Spermatids show maturation defects during the stages of spermiogenesis, characterized by premature degeneration of spermatids, apoptosis, sloughing into the lumen, and few spermatids produced in the functional lumen that exhibit malformed heads with disorganized alignment. Most importantly, PD‐L1 overexpression in the seminiferous and Sertoli cells of the seminiferous tubules only caused spermatid release disorder. Therefore, the results obtained from the study of PD‐L1 in Sertoli and germ cells may provide us with an investigational basis regarding the potential regulatory mechanisms of PD‐L1 in affecting cell adhesion function.

## 2 MATERIALS AND METHODS

### 2.1 Animals

Experimental animal materials included male and female C57BL/6 (B6) mice (purchased from the Shanghai Sleek Experimental Animal Centre). All experimental procedures were approved by the Institutional Animal Care and Use Committee of South China University of Technology. Transgenic mice and wild‐type mice were fed in an SPF‐grade animal laboratory and sacrificed at 22, 40, 60, 70, 90, 100, 120, 140, 160 and 190 days. At each time point, wild‐type mice and transgenic mice were divided into WT and PD‐L1 groups, with five mice in each group. Testis and epididymis were immediately taken, photographed and weighed, and the average weight of testicular and epididymal tissues of mice was calculated.

### 2.2 Construction of the transgenic vectors

**FIGURE 1:**
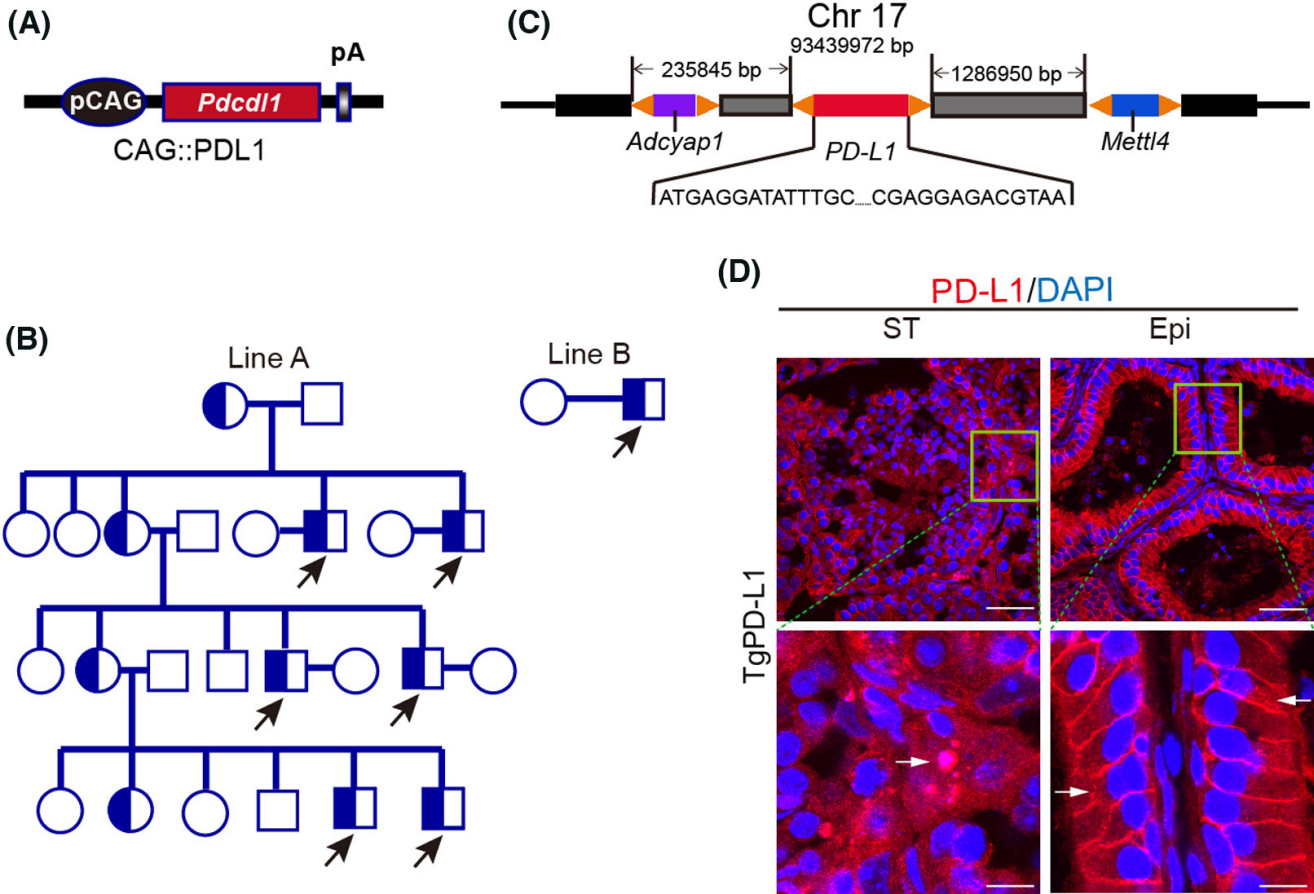
PD‐L1 transgenic male mice develop infertility. (A) Overexpression plasmid for exogenous PD‐L1. (B) Breeding lineage diagram of PD‐L1 transgenic mice (circles indicate females, boxes represent males and arrowheads indicate PD‐L1 transgenic male mice, all infertile). (C) Whole genome sequencing analysis of PD‐L1 transgenic mice for gene insertion location. (D) Immunofluorescence staining of the seminiferous tubules (ST) and epididymal duct (Epi) using PD‐L1 (red) and DAPI (blue) in PD‐L1 transgenic mice. Arrows indicate PD‐L1‐positive germ cells. Scale bars: 50 μm (top); 10 um (bottom)

PD‐L1 transgenic mice were generated using CAG‐PD‐L1 and CAG‐PD‐L1‐IRES2‐EGFP vectors respectively.^28^ First, total RNA was isolated from the spleen of mice, and cDNA for PD‐L1 was generated by PCR using the following primers: sense, 5′‐CGACTCGAGATGAGGATATTTGC‐3′ and antisense, 5′‐CAGGAATTCTTACGTCTCCTCGA‐3′ (Shenggong Co. Shanghai, China). The cDNA was cloned into the TA cloning vector pMD18‐T and was inserted between the *Xho* I and *EcoR* I sites of pCAGGS (a gift from Dr. Miyazaki J) to construct the pCAG‐PD‐L1 plasmid. The pCAG‐PD‐L1 plasmid was digested with *Sal* I and *Avr* II, and linearized CAG‐PD‐L1 transgenic fragments were collected and prepared for pronuclear microinjection of fertilized eggs (Figure 1A).

Next, PD‐L1 cDNA was cloned into pIRES2‐EGFP (Clontech Co. USA) to construct the pPD‐L1‐IRES2‐EGFP plasmid. The pPD‐L1‐IRES2‐EGFP plasmid was digested with *Xho* I (10 units/µg DNA, R0146S, New England Biolabs, USA) and *EcoR* I (10 units/µg DNA, R0101S, New England Biolabs, USA) restriction enzymes to liberate PD‐L1‐IRES2‐EGFP fragments, which were then subcloned into the pCAGGS vector to construct the pCAG‐PD‐L1‐IRES2‐EGFP vector. We linearized this vector using *Spe* I (10 units/µg DNA, R0133S, New England Biolabs, USA) and *Ssp* I (10 units/µg DNA, R0132S, New England Biolabs, USA) prior to pronuclear microinjection of fertilized eggs (Figure 4A). Similarly, PD‐L1 cDNA was cloned into the Prmt‐EGFP vector to construct the Prmt‐PD‐L1‐EGFP plasmid (Figure 4D).

### 2.3 Generation of PD‐L1 transgenic mice

Superovulation procedures were performed following the previously described procedures.^29^ Briefly, 4‐week‐old (C57BL/6) female mice were superovulated by intraperitoneal injection of 5 IU pregnant mare serum gonadotropin (PMSG, Sigma–Aldrich, St. Louis, MO, USA), followed 48 h later by 5 IU of human chorionic gonadotropin. The mice were then crossed with 8‐week‐old (C57BL/6) male mice. The PD‐L1 transgenic fragments were diluted to 1 ng/ml in microinjection buffer (5 mM Tris, 0.1 mM EDTA, pH 7.4) and microinjected into the male pronuclei of embryos, which were cultured for 1 day in a microdrop of M16 medium (MR016, Sigma–Aldrich, USA). All solutions used for culture experiments were pre‐equilibrated at 37°C and 5% CO_2_ and covered with mineral oil (M5904, Sigma–Aldrich, USA). Two‐cell embryos were picked up and transferred to pseudopregnant ICR foster mothers. Positive PD‐L1 transgenic mice were detected by PCR using specific primers (sense, GTGATTCAGTTTGTGGCAGGAG; antisense, ACCGTGGACACTACAATGAGGA).

### 2.4 Periodic acid‐Schiff (PAS) Staining

Periodic acid‐Schiff staining was performed as previously reported.^30^ After organ extraction, fix mouse testes and epididymis in Bouin's fixative solution (PH0976, Phygene, China) for 24 h at room temperature and then put in 70% ethanol, then embed testis and epididymis in paraffin (76242, Sigma–Aldrich, USA). Paraffin‐embedded mouse testis and epididymis tissues were cut into 5 μm thick testis slices and mount on normal glass slides. The slices were deparaffinized in xylene (LM1330‐20–7, LMAI Bio, China), hydrated in gradient alcohol (100%, 95%, 80% and 70%), immersed in iodic acid oxidation solution (77310, Sigma–Aldrich, USA) for 5 min and in Schiff reagent (1.09033, Sigma–Aldrich, USA) for 15 min and counterstained with haematoxylin (H9627, Sigma–Aldrich, USA) for 3 min. Wash slides in running distilled water for 15 min, then dehydrate in alcohol and clear in xylene. The sections were photographed with an optical microscope (Leica, Germany).

### 2.5 Haematoxylin‐Eosin (HE) Staining

Haematoxylin‐Eosin staining was performed as previously reported.^7^ Fix mouse testes and epididymis in Bouin's fixative solution (PH0976, Phygene, China) for 24 h at room temperature and then put in 70% ethanol, then embed testis and epididymis in paraffin (76242, Sigma–Aldrich, USA). Paraffin‐embedded mouse testis and epididymis tissues were cut into 5 μm thick testis slices and mount on normal glass slides. Then, the slices were deparaffinized in xylene (LM1330‐20‐7, LMAI Bio, China), hydrated in gradient alcohol (100%, 95%, 80%, and 70%) and wash slides in running distilled water for 2 min, then stained with haematoxylin and eosin (C0105M, Beyotime, China). The sections were photographed with an optical microscope (Leica, Germany).

### 2.6 Immunofluorescence (IF)

For IF, place paraffin slices in 75°C for half an hour to prevent slices from falling off, dewaxing and hydration. Then, tissue sections were placed vertically in Tris‐EDTA antigen repair solution (ST725, Beyotime, China) and heated for 10 min at 65°C to repair the antigens, and removed to room temperature. After that, blocked it with 10% goat serum (G9023, Sigma–Aldrich, USA) for 1 h at room temperature, and then incubated with DAPI/PI/anti‐PD‐L1 antibodies (DAPI, 5 mg/ml, C1002, Beyotime, China; PI, 5 mg/ml, P0135, Beyotime, China; Anti‐PD‐L1, 1:250, 66248‐1‐Ig, Proteintech, China) overnight at 4°C. After washing with 1× TBST (ST673, Beyotime, China) for 5 min, the sections were incubated with the appropriate secondary antibody (488‐conjugated antibody, 1:500, 115‐545‐146; 680‐conjugated antibody, 1:500, 115‐625‐146, Jackson ImmunoResearch Laboratories, USA) for 30 min at room temperature. For the negative control, sections were incubated with the secondary antibody only. Further detection was done by following the manufacture protocol. Photographs were taken under the same conditions with a fluorescence microscope (Leica, Germany). The experiment was repeated three times.

### 2.7 Integrity test of the blood‐testicular barrier

Both wild‐type mice and transgenic mice were adult mice of the same age. The wild‐type mice in the positive control group were given 3 mg/kg CdCl_2_ (202908, Merck, Germany), while the wild‐type mice in the negative control group and the transgenic mice in the experimental group were injected with the same volume of normal saline. Wild‐type mice and transgenic mice were divided into CdCl2, WT and TgPD‐L1 groups, with five mice in each group. Ten milligrams of FITC‐lectin (10 µg/ml, L32470, Thermo Fisher, USA) was injected into the tail vein after 24 h. After 20 min, the mice were euthanized and the testis were removed via abdominal incision. Tissues were fixed overnight with 4% paraformaldehyde, and then dehydrated with 25% sucrose solution. Finally, 12‐μm sections were mounted onto glass slides for observation with a fluorescence microscope (Leica, Germany).

### 2.8 Parabiosis test

We used TgGFP and TgPD‐L1 transgenic male mice for our parabiosis test using the following procedure that was adopted from published methods.^31^ Five TgGFP mice and five TgPD‐L1 transgenic mice were used in the parabiosis test. Two paired mice were anaesthetized using an injectable anaesthetic (sodium pentobarbital, 40–60 mg/kg, intra‐Pperitoneal). A longitudinal skin incision was made from 0.5 cm above the elbow all the way to 0.5 cm below the knee joint using sharp scissors on the prepared side of each animal. The skin along the incision was gently separated from subcutaneous fascia using forceps. The right elbow and knee of the animal on the left were joined to the left elbow and knee of the animal on the right, respectively, using 2–0/3–0 silk sutures. The incision was closed with simple interrupted sutures on both the ventral and dorsal sides. Blood chimaerism usually occurs approximately 2 weeks following surgery. At the end of the experiment, the mice were euthanized to separate the pairs to collect testis tissues and plasma. The parabiosis experiment was repeated twice for a total of three experiments.

### 2.9 Cell culture

The human colorectal cancer cell line SW480 and the human embryonic kidney 293T cell line were purchased from ATCC (MA, USA). The HEK293T cell line was cultured in DMEM (C11995500BT, Gibco, USA) supplemented with 10% FBS (10099141, Gibco, USA) and 1% penicillin‐streptomycin solution (C0222, Beyotime, China). The SW480 cell line was cultured in RPMI 1640 medium (C11875500, Gibco, USA) supplemented with 10% FBS and 1% penicillin‐streptomycin solution. All cell lines were cultured at 5% CO_2_ and 37°C.

### 2.10 Lentivirus production and infection

Full‐length human PD‐L1‐coding cDNA with HA and Flag labels was PCR amplified from CS‐U0767‐pIRES and CS‐U0767‐M83 (purchased from GeneCopoeia Company, China). Primers for PCR are in Table S1. All lentiviruses were packaged in 293T cells with psPAX2 (#12260, Addgene, USA) and pMD2.G (#12259, Addgene, USA), purified and concentrated using 0.45 μm syringe filters (4614, Pall Corporation, USA), and then aliquoted and stored at −80°C. Cells were infected with concentrated lentivirus solution at a final concentration of 1/50 and screened with 3 μg/ml purinomycin (A1113803, Thermo Fisher, USA).

### 2.11 RNA extraction, RT‐PCR and qRT‐PCR

Total RNA was extracted with TRIzol reagent (TIANGEN, China) followed by RaPure Cell RNA Mini Kit (4010, Magen, China) treatment using the manufacturer's protocol and transcribed into cDNA by using PrimeScript™ RT Master Mix (RR036A, TaKaRa, Japan) according to the manufacturer's instructions (37°C, 15 min for reverse‐transcription, 85°C, 5 s for heat inactivation of reverse transcriptase, 4°C end).

For reverse transcription‐PCR (RT‐PCR), cDNA was then amplified using gene‐specific primers designed using NCBI primer design tool (https://www.ncbi.nlm.nih.gov/tools/primer‐blast/). Primer sequences are listed in Table S1. The RT‐PCR condition was 35 cycles of 95°C for 30 s, 60°C for 30 s and 72°C for 30 s, followed by a 10 min extension at 72°C with GoTaq DNA polymerase (R004A, TaKaRa, Japna), which lacks 5′ to 3′ exonuclease activity.

For quantitative reverse transcriptase‐PCR (qRT‐PCR), the cDNA was diluted 1:50 and qRT‐PCR assays were performed by using the SYBR^®^ Green Premix kit (639676, TaKaRa, Japan) according to the manufacturer's protocols. Reactions were run on an LightCycler^®^ 96 System (Roche, Switzerland) and samples were analysed in triplicates. The qRT‐PCR cycling conditions were 95°C for 10 s, 58°C for 20 s and 72°C for 15 s. Melt curve data were obtained to confirm amplification of the correct product in each well. *Gapdh* was used as an internal reference gene, and all data analyses were performed using the comparative Ct method. Primers for PCR are shown in Table S1. The experiments were repeated three times.

### 2.12 Co‐Immunoprecipitation assay

The cells were collected by a cell scraper (Corning, USA) with cool lysate (Beyotime, China) for protein extraction. The lysate was incubated with anti‐Flag antibodies (F1804, Merck, Germany) or an equal amount of mouse IgG (B900620, Proteintech, China) on a rotating wheel overnight at 4°C and then incubated with protein A agarose beads (Beyotime, China) at 4°C for 10 h. Beads were collected by centrifugation, washed, boiled in 2× PAGE loading buffers (Beyotime, China) and analysed by Western blotting. The experiments were repeated three times.

### 2.13 Western blotting analysis

The protein was separated by 10% SDS‐PAGE and transferred onto polyvinylidene difluoride (PVDF) membranes (Merck Millipore, Ireland). The membranes were blotted with anti‐HA antibodies (ab9110, Abcam, UK), PD‐L1 (ab205921, Abcam, UK), anti‐GAPDH antibodies (bs‐2188r, Bioss, China) and secondary antibodies (ARG24083 and ARG65350, Arigobio, China) to detect the proteins. GAPDH protein was used as a loading control. The experiments were repeated three times.

### 2.14 Statistical analysis

All experiments were repeated independently at least three times. Statistical analysis was performed using GraphPad Prism Software. Quantitative data are displayed as the mean ± SEM in each experiment. Comparisons among multiple groups were performed through one‐way ANOVA with Newman–Keuls post‐test. *p* < 0.05 (two‐tailed) was considered statistically significant.

## 3 RESULT

### 3.1 Overexpression of PD‐L1 leads to infertility in male mice

PD‐L1 (programmed cell death ligand‐1), also known as B7‐H1, was initially reported to be involved in the negative regulation of cell‐mediated immune responses as a B7 family member and was later found to be one of the ligands acting as PD‐1 (programmed cell death‐1).^14^, ^32^ To further explore the unknown physiological function of PD‐L1 in vivo, we followed a strategy of generating PD‐L1 transgenic (TgPD‐L1) mice. We first constructed a PD‐L1 (mice) overexpression plasmid with the *Pdcdl1* gene under the CAGGS (CAG) promoter to generate TgPD‐L1 mice by fertilized egg microinjection^33^ (Figure 1A). We obtained two lines of TgPD‐L1 founder mice, Line A and Line B (Figure 1B). Next, we crossed Line A and Line B TgPD‐L1 mice with wild‐type (WT) mice. Linage analysis showed that TgPD‐L1 male mice were infertile, while TgPD‐L1 female mice exhibited normal fertility and productivity (Figure 1B). Whole‐genome sequencing of Line A showed that the exogenous *Pdcdl1* gene was inserted at 93,439,972 bp on mouse chromosome 17 without inserting into known genes in mice^34^ (Figure 1C). PD‐L1 was overexpressed in cells of seminiferous tubules, including spermatids and Sertoli cells, and the epididymis was assessed by immunofluorescence (IF) staining (Figure 1D and Figure S1C). These results showed that excessive PD‐L1 can cause infertility in male but not in female mice. This suggests that high expression of PD‐L1 in the testis may lead to infertility in mice.

### 3.2 Seminiferous epithelium of PD‐L1 transgenic mice exhibits germ cells sloughing

**FIGURE 2:**
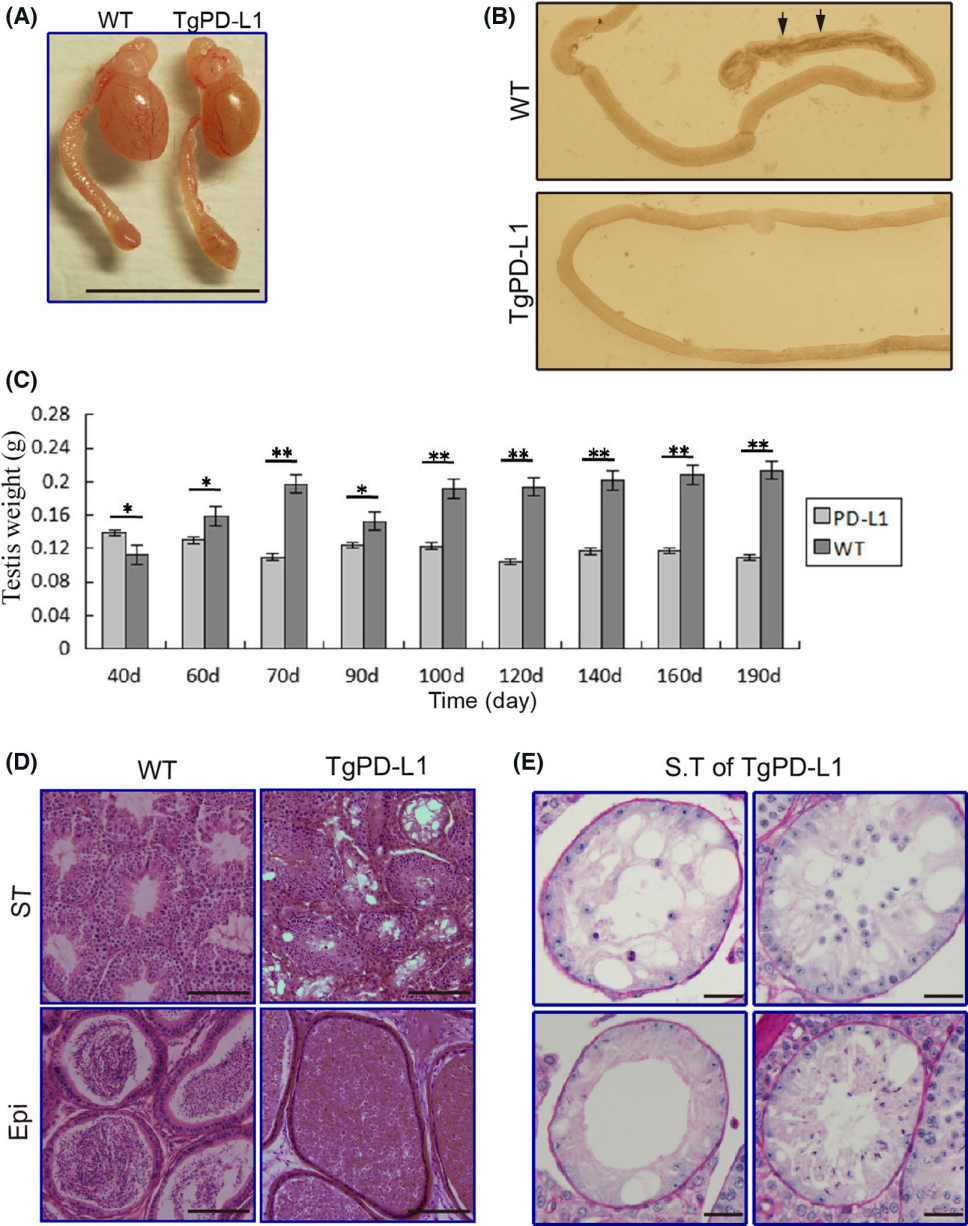
Testicular abnormalities in PD‐L1 transgenic mice. (A) Size of testes from wild type (WT) and PD‐L1 transgenic mice. Scale bars, 1 cm. (B) Illumination test of the epididymal ducts from WT and PD‐L1 transgenic mice (arrowheads indicate sperm cell clumps). (C) Time course of analysis of weight of testes of WT and PD‐L1 transgenic mice from 40 to 190 days. Data represent the mean ± SD of 3 replicates. *p < 0.05, **p < 0.01. (D) HE staining of seminiferous tubules (ST) and epididymal duct (Epi) from WT, and TgPD‐L1 mice. Scale bars, 200 μm. (E) PAS staining of seminiferous tubules (ST) from PD‐L1 transgenic mice. Scale bars, 40 μm

The testis size of TgPD‐L1 was much smaller than that of WT mice (Figure 2A). Compared with that of WT mice, the seminiferous tubules lacked dark areas in TgPD‐L1 mice, where mature sperm were released from the seminiferous epithelium and accumulated in the lumen of the seminiferous tubules by transilluminated observation (Figure 2B). The weights of the body and epididymis were not different between TgPD‐L1 and WT mice (Figure S1). However, the testicular weight was obviously decreased at approximately 60 days of age during the 190‐day observation period in TgPD‐L1 mice, even though the testes were more weight‐intensive in TgPD‐L1 mice than in WT mice at approximately 40 days after birth (Figure 2C). The epithelium of seminiferous tubules was arranged in a disorderly manner, and many cells were lost in TgPD‐L1 mice, as shown in testis cross sections by HE staining (Figure 2D). Cross sections of seminiferous tubules with PAS staining showed that spermatocytes were lost in different stages during their development, with Sertoli cells remaining in TgPD‐L1 mice (Figure 2E). Compared with the mature sperm that filled the epididymal lumen in WT mice, there were few mature sperm with many sloughing round spermatids in TgPD‐L1 mice (Figure 2D). These results suggest that obstacles in spermatogenesis are present with the sloughing of spermatocytes from the seminiferous epithelium in TgPD‐L1 mice.

### 3.3 Spermatogenesis was disordered with gradual severe sloughing of testicular cells after sexual maturation in TgPD‐L1 mice

**FIGURE 3:**
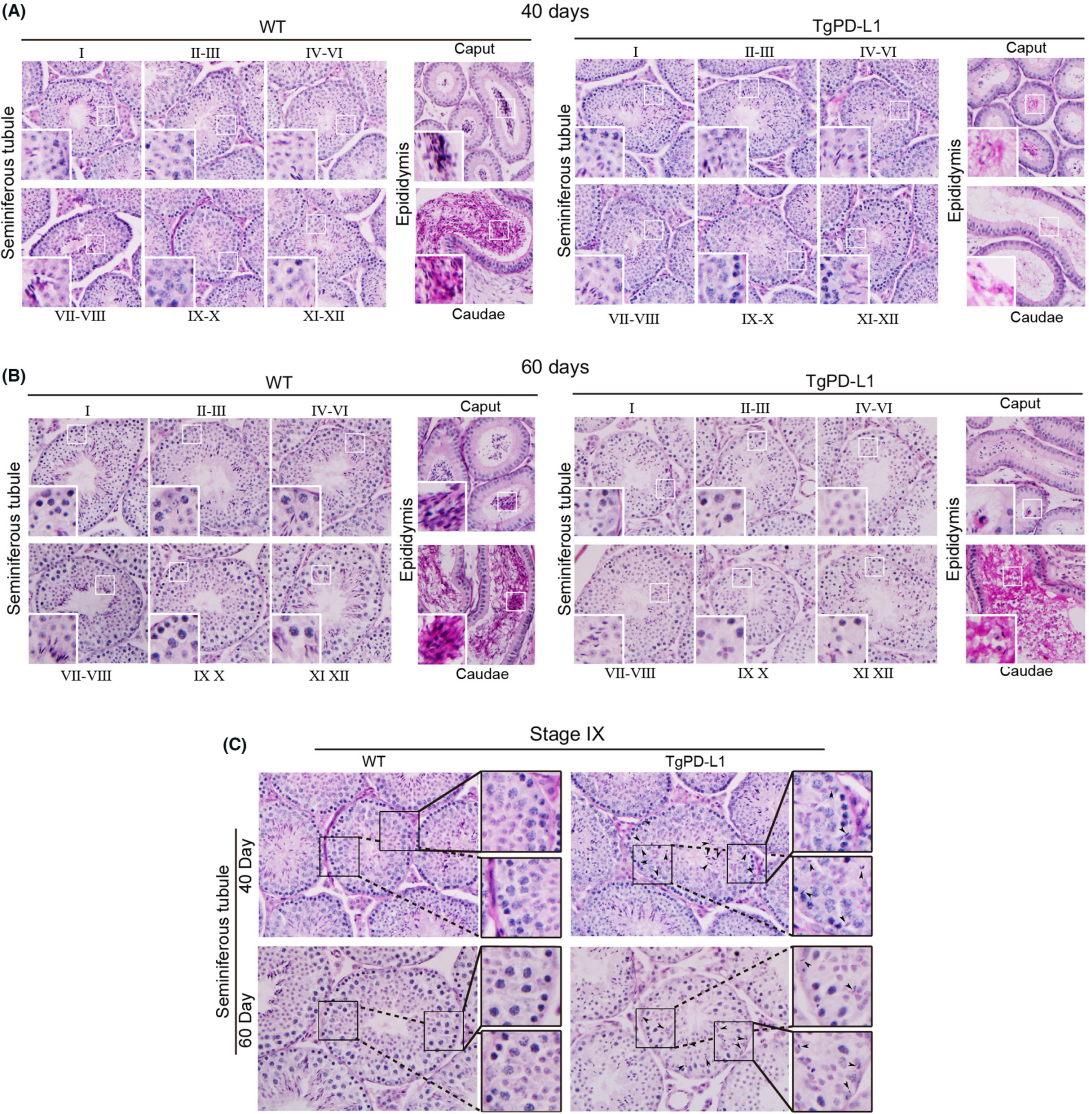
Spermatogenesis was disordered after sexually maturation in TgPD‐L1 mice. (A) PAS staining was performed to analyse the spermatid development from seminiferous tubules to epididymal tubules at different stages (I to XII) on Day 40 and Day 60 (B) Magnification, 400×. Boxed areas are enlarged in the bottom left corner. (C) PAS staining confirmed spermatid (stage Ⅸ) development and spermiation failure in the mouse seminiferous epithelium (day 40 and 60). Areas outlined by squares are enlarged on the right. Magnification, 400× (left) and 1000× (right)

The stages in the cycle of seminiferous epithelium and developmental steps of germ cells were evaluated via cross sections of testes in WT and TgPD‐L1 mice. At 22 days of age, there were no distinguished differences in histological observation by PAS staining between WT and TgPD‐L1 mice (Figure S2). At approximately 40 days of age, male mice are sexually mature and experience the first wave of sperm release from the seminiferous tubules to the epididymis lumen.^7^ At this time point, the histological structure of the seminiferous epithelium was intact without obvious sloughing of germ cells (Figure 3A). The stages of the cycle of seminiferous epithelium and the steps of germ cell development could be easily recognized in both the testes of WT and TgPD‐L1 mice (Figure 3A). Some mature sperm were found in the caput and caudae of the epididymis in TgPD‐L1 mice, but far fewer than those in WT mice (Figure 3A). However, at the age of 60 days, the structure of the seminiferous epithelium was obviously disordered, with loose cell arrangement, and lacked the VI step of germ cell development followed by spermiation at the VII‐VIII stage in the seminiferous epithelium cycle in TgPD‐L1 mice (Figure 3B). At this time point, few mature sperm but no sloughed round spermatids accumulated in the caput and caudae of the epididymis in TgPD‐L1 mice, while full mature sperm could be seen in the epididymal lumen in WT mice (Figure 3B). At the Ⅸ stage of the seminiferous epithelium cycle after spermiation, highly condensed nuclei of elongated spermatids were drawn to the basement of seminiferous tubules in TgPD‐L1 but not in WT testes (Figure 3C). This phenotype is a typical feature of spermiation failure in processes of germ cell development.^6^ All these results showed that spermiation failure and testicular cell sloughing caused spermatogenesis disorders to induce infertility in TgPD‐L1 mice.

### 3.4 Only PD‐L1 overexpression on both Sertoli cells and spermatids of the seminiferous epithelium causes sloughing of testicular cells

**FIGURE 4:**
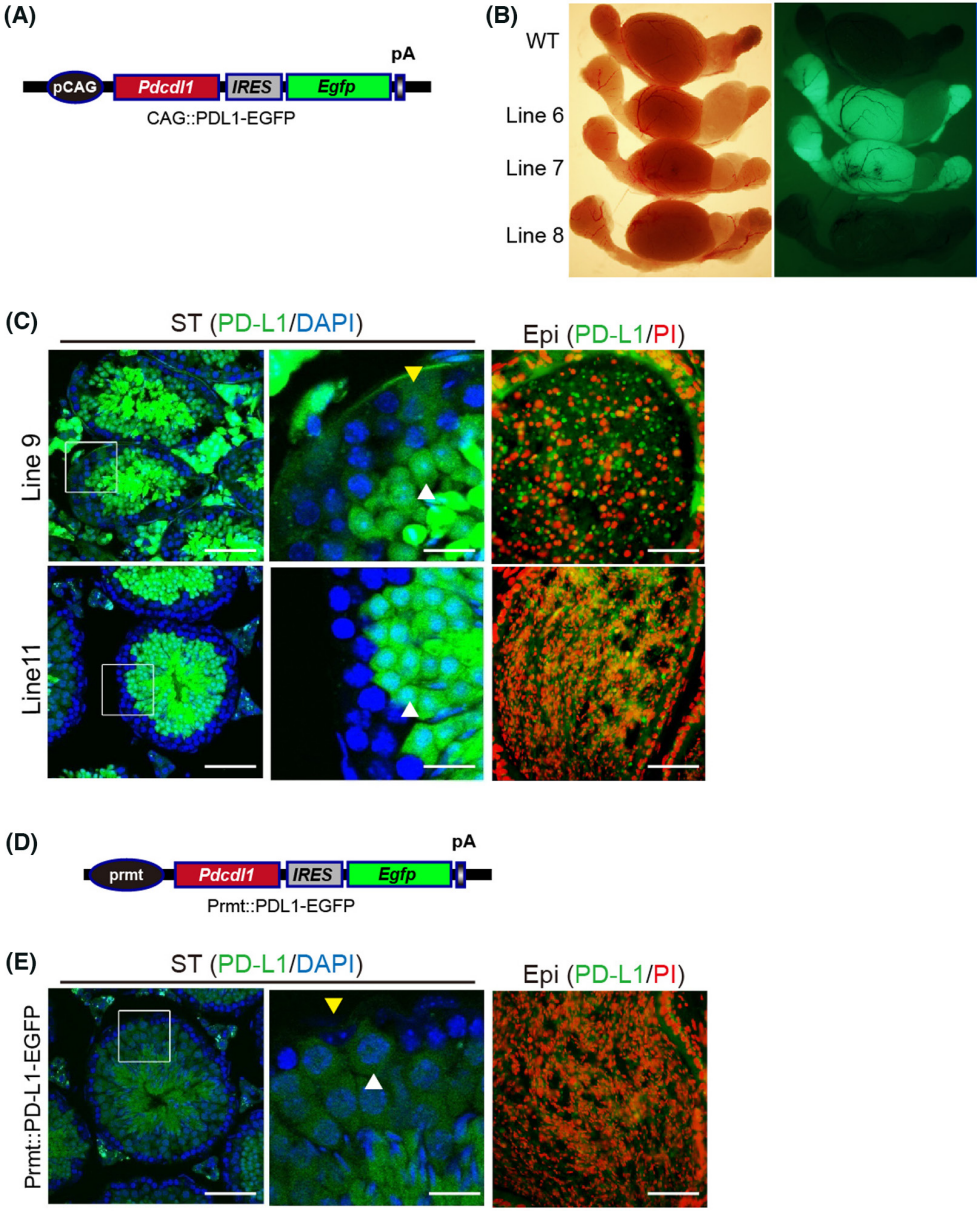
Only PD‐L1 overexpression on both Sertoli cells and spermatids of the seminiferous epithelium causes sloughing of testicular cells. (A) Overexpression plasmid for exogenous PD‐L1 with enhanced green fluorescent protein (EGFP). (B) Transfection test in testis and epididymis of WT and PD‐L1 transgenic mice (Line 6, Line 7, Line 8). (C) Immunofluorescence staining of the seminiferous tubules (ST) and epididymal duct (Epi) using PD‐L1 (green), DAPI (blue) and PI (red) in line 9, line 11 and Prmt::PD‐L1‐EGFP (E) transgenic mice. Arrowheads indicate Sertoli cells (yellow) and spermatocytes (white). Scale bars, 50 μm (left and right); 10μm (middle). (D) Plasmid overexpressing PD‐L1 and EGFP specifically in spermatogonia

To further confirm that high expression of PD‐L1 in the testis causes infertility in mice and exclude the possibility of insertional mutations during the transgenic processes, we generated several other lines of TgPD‐L1 mice with *Egfp* reporter genes under the CAG promoter (TgPD‐L1‐EGFP) (Figure 4A). We obtained 11 other lines of PD‐L1 transgenic founder mice by PCR detection (Table 1). Only testes with high EGFP (EGFP+) expression in mice were infertile, while EGFP‐ testes were productive with normal fertility (Figure 4B and Table 1). These results suggest that only testes with high PD‐L1 expression can cause infertility in mice. These results also excluded the possibility that exogenous PD‐L1 caused insertional mutations leading to infertility by transgenic processes in male mice.

**TABLE 1:** Fertility of PD‐L1 founders and offsprings

Line	Sex of founder	Fertility in founder	EGFP in testes of founder	Fertility in male offspring	EGFP in testes of offspring
1	M	+	−	+	−
2	M	+	−	+	−
3	F	+	/	−	+
4	M	+	−	+	−
5	M	+	−	+	−
6	M	−	+	−	/
7	M	−	+	−	/
8	M	+	−	+	−
9	F	+	/	−	+
10	F	+	/	−	+
11	F	+	/	+	+

Abbreviations: −, infertility; /, none; +, fertility; F, female; M, male.

However, we also found that Line11 mice with high expression of EGFP in testes could normally be fertile (Table 1). Therefore, we chose Line 9 and Line 11 mice to detect the reasons why both EGFP+testes caused different fertility in TgPD‐L1 mice. In Line 9 mice, sloughed round spermatids were found in the epididymis with few mature sperms (Figure 4C, upper panel). Both spermatocytes and Sertoli cells highly expressed EGFP in cross sections of seminiferous tubules in Line 9 mice under observation by fluorescence microscopy (Figure 4C, upper panel). We found full mature sperms in the epididymis in Line 11 mice with few sloughed round spermatids (Figure 4C, lower panel). Most importantly, only spermatids but not Sertoli cells had high EGFP expression in the seminiferous tubules of Line 11 (Figure 4C, lower panel). These results strongly suggest that both Sertoli cells and spermatids expressing PD‐L1 are necessary for infertility in TgPD‐L1 mice.

To further confirm the hypothesis that both Sertoli cells and spermatid cells are needed to express PD‐L1 to induce spermatid sloughing and infertility in TgPD‐L1 mice, we constructed another transgenic vector that coexpressed PD‐L1 and EGFP under the promoter of protamine 1 (P1), which was shown to be translated at spermatid stages during development^35^ (Figure 4D). The seminiferous epithelium was intact, and elongating spermatids showed higher EGFP expression than spermatocytes and round spermatids in Prmt::PD‐L1‐EGFP mice (Figure 4E). There were full sperm in the epididymis without sloughing spermatids and normal fertility in Prmt::PD‐L1‐EGFP mice (Figure 4E). These results confirmed that simultaneous expression of PD‐L1 in both Sertoli cells and spermatids can cause cell sloughing in the seminiferous epithelium and infertility in male mice.

### 3.5 Ruling out the possibility that PD‐L1 and other molecular effects contribute to germ cell sloughing in the seminiferous tubules

**FIGURE 5:**
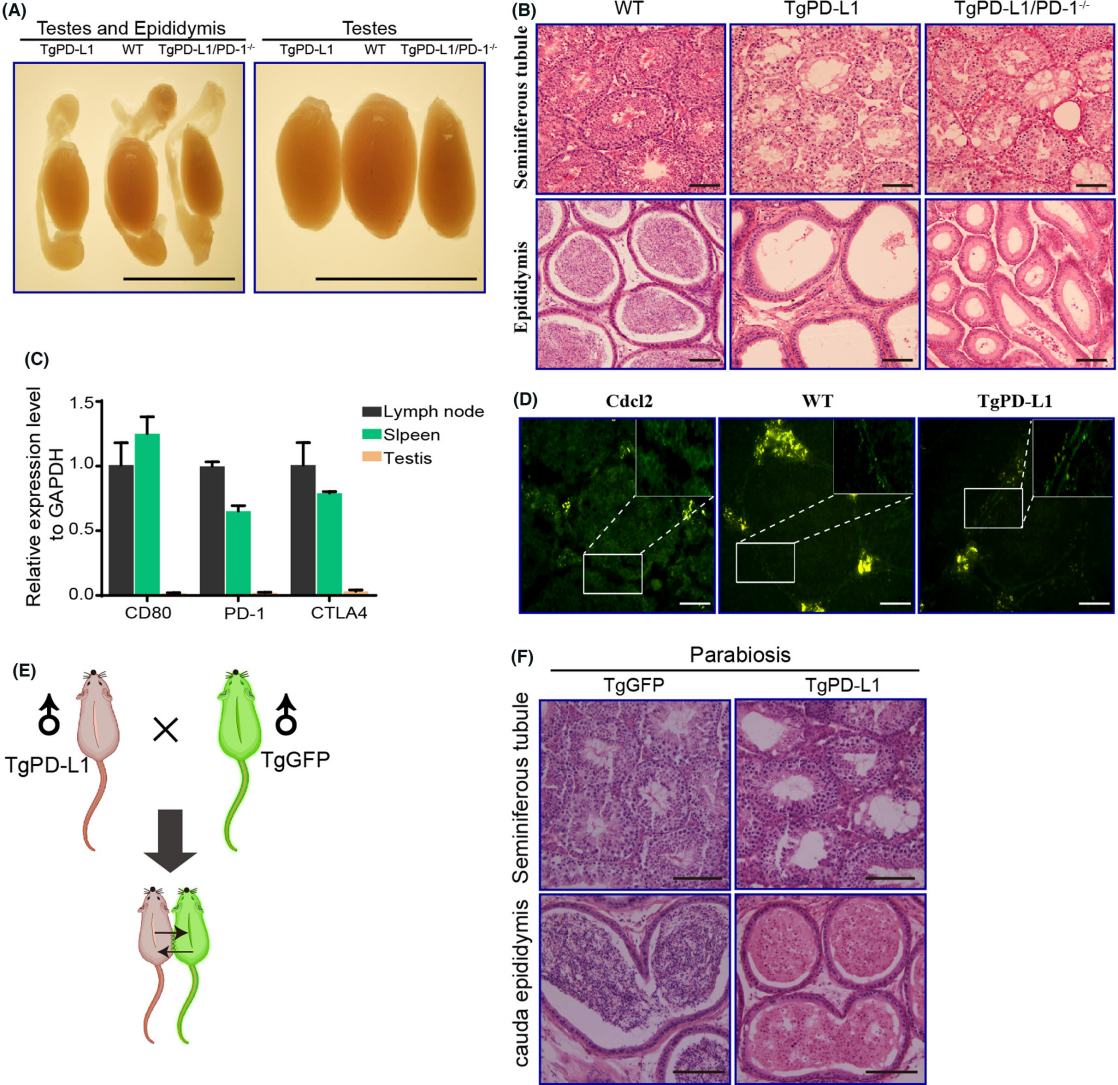
Rule out the possibility that PD‐L1 and other molecular effects contribute to spermatids sloughing in the seminiferous tubules. (A) Size of testes from WT, PD‐L1 and PD‐L1/PD‐1^−/−^ transgenic mice. Scale bars, 1 cm. (B) PAS staining of seminiferous tubules (ST) and epididymal duct (Epi) from WT, PD‐L1 and PD‐L1/PD‐1^−/−^ transgenic mice. Scale bars, 100 μm. (C) mRNA levels of CD80, PD‐1 and CTLA4 were determined by quantitative RT‐PCR in lymph nodes, spleens and testicular tissues of transgenic mice. (D) CdCl_2_ experiments confirmed that the blood testis barrier of seminiferous tubules was not disrupted in PD‐L1 transgenic mice. Scale bars, 50 μm. Boxed areas are magnified in the top right corner. (E) Flowchart of skin suture experiments. (F) HE staining of seminiferous tubules (ST) and epididymal duct (Epi) from TgEGFP and TgPD‐L1 mice. Scale bars, 200 μm

We wanted to further confirm the specific mechanism by which PD‐L1 caused germ cell sloughing. PD‐1 is the main receptor that binds PD‐L1 to transduce immunosuppressive signals.^16^ If the infertility of male TgPD‐L1 mice is due to PD‐1/PD‐L1 binding, blocking this pathway can rescue the infertility of male TgPD‐L1 mice. Therefore, we crossed female TgPD‐L1 mice with male PD‐1 knockout (PD‐1^−/−^) mice to produce TgPD‐L1/PD‐1^−/−^ mice. Unfortunately, TgPD‐L1/PD‐1^−/−^ mice were still infertile, with smaller testes, germ cells sloughing in the seminiferous epithelium and no mature sperm in the epididymis (Figure 5A,B). RT‐PCR detection also confirmed that the transcripts of PD‐1, CD80 and CTLA‐4 were expressed at low levels in the testis compared with the spleen and lymph nodes in WT mice (Figure 5C). These results suggested that signalling of PD‐L1 overexpression in the testis caused germ cell sloughing and that infertility might not occur through the PD‐L1/PD‐1 or PD‐L1/CD80 pathways. The special histological structure of spermatids only contacts Sertoli cells during germ cell development in the testis.^11^, ^27^ We hypothesized that PD‐L1 expressed on Sertoli cells and spermatids could bind each other to cause signal transduction and induce germ cell sloughing from the seminiferous epithelium and infertility in male mice. To exclude other factors that cause infertility in male mice, we first detected the intact blood testis barrier (BTB). Disruption of the BTB can interfere with spermatogenesis and thereby cause spermatid sloughing.^36^, ^37^ We found that the BTB remained intact in TgPD‐L1 mice using intraperitoneal injection of CdCl_2_ (Figure 5D). Next, we analysed how humoral factors influence the infertility of male mice by parabiosis via TgPD‐L1 (Line A) by suturing with TgGFP mice (Figure 5E). Neither TgPD‐L1 nor TgGFP mice influenced the structures of the seminiferous epithelium and epididymis of their parabiotic partners (Figure 5F and Figure S3). The parabiotic TgPD‐L1 and TgGFP mice were separated after 1.5 months of parabiosis, and both maintained infertility and fertility without any influence. We performed whole genome sequencing of the transgenic mice and established that multiple lines shared the same phenotype, thus excluding the possibility of an insertion mutation. Therefore, the above results strongly confirmed that PD‐L1/PD‐L1 signalling caused defects in spermatogenesis in TgPD‐L1 male mice.

### 3.6 PD‐L1 can bind PD‐L1

**FIGURE 6:**
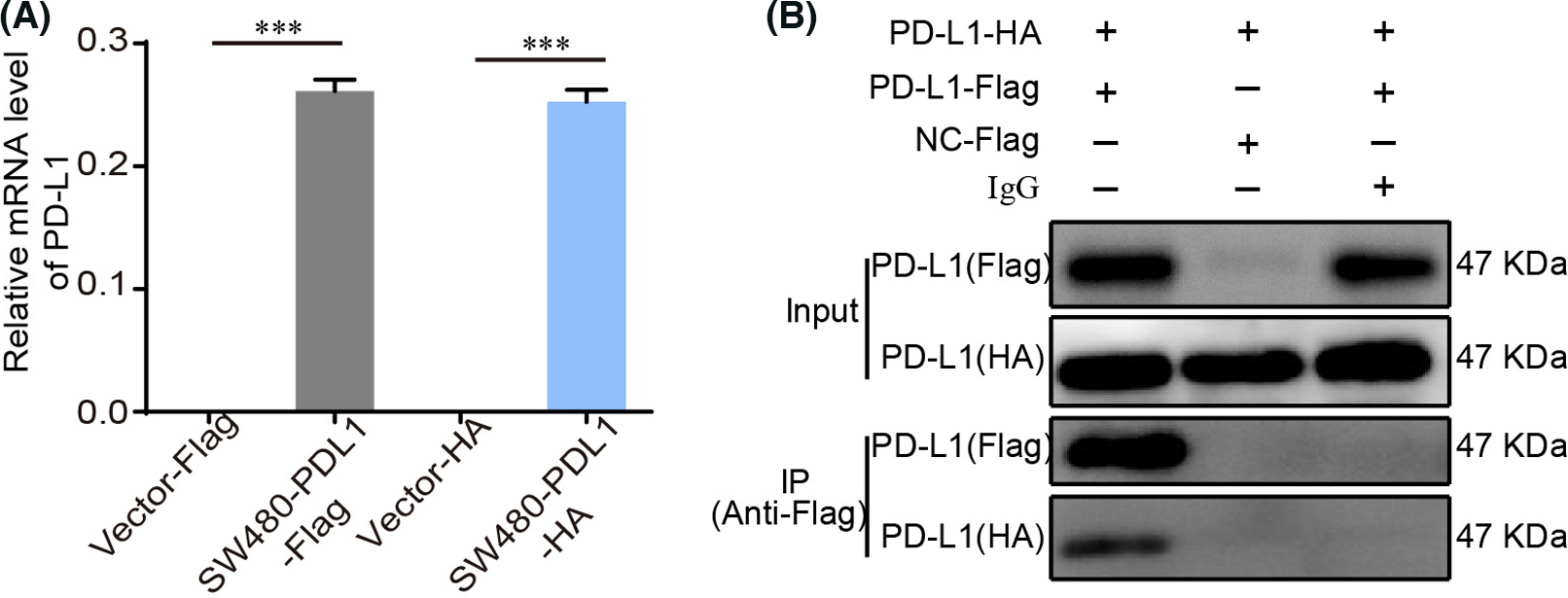
PD‐L1 can bind PD‐L1. (A) mRNA levels of PD‐L1 was determined by quantitative RT‐PCR in Vector‐Flag, SW480‐PD‐L1‐Flag, Vector‐HA and SW480‐PD‐L1‐HA cells. Data represent the mean ± SD of 3 replicates. ***p < 0.001. (B) Co‐IP analysis of interactions between SW480‐Flag and SW480‐HA

We further confirmed the hypothesis that PD‐L1 can function by binding to PD‐L1 on other cells. First, we constructed PD‐L1 overexpression vectors with different tags and transfected them into SW480 cells to obtain the stable PD‐L1 overexpression cell lines sw480‐PD‐L1‐flag and sw480‐PD‐L1‐HA (Figure 6A). Coimmunoprecipitation (co‐IP) revealed an interaction between PD‐L1 and PD‐L1 molecules (Figure 6B). This result proved our hypothesis that PD‐L1 can bind to PD‐L1 and transduce signals to have biological effects in mice.

## 4 DISCUSSION

PD‐L1, also known as B7‐H1, was originally reported in 1999 as a B7 family member involved in the negative regulation of cell‐mediated immune responses.^14^ PD‐1/PD‐L1 is an important immune ‘checkpoint’ signalling pathway that plays a fundamental role in tumour immunity and has also become a focus of recent research.^38^, ^39^ PD‐L1 is a type I transmembrane protein containing an IGV and IGC ectodomain in the extracellular region linked to an intracellular domain by a hydrophobic transmembrane domain. This intracellular domain is not found to contain a classical signalling domain,^40^ which is one of the reasons why PD‐L1 is not thought to have a cell signalling function. The physiological function of PD‐L1 and its mechanism of action in various diseases are poorly defined. Therefore, we constructed PD‐L1 transgenic mice for exploration. In the present study, overexpression of PD‐L1 caused abnormal testicular shrinkage and male infertility in mice, which were associated with abnormalities in spermatogenesis. Furthermore, we uncovered a critical role for PD‐L1 in causing these abnormalities.

PD‐1/PD‐L1 has recently been identified as an important negative regulatory signalling pathway in immunity.^41^ The interaction of PD‐1 and its ligand PD‐L1 provide a bidirectional regulatory signal for lymphocyte activation and promote immune activation and immune tolerance, thereby regulating immune responses. Studies have confirmed that PD‐1 is a negative regulatory molecule of T cells.^42^ In addition to PD‐1, studies have shown that PD‐L1 and the costimulatory protein CD80 interact strongly in cis rather than in trans at the cell membrane, and further studies showed that the PD‐L1‐CD80 cis interaction not only inhibited the trans interaction between PD‐L1 and PD‐1 but also reduced PD‐1/PD‐L1 signalling and CD80‐CTLA4 binding.^17^ Receptors PD‐1 and CD80 play a key role in the negative regulation of cell‐mediated immune responses.^16^, ^17^, ^43^ However, PD‐1 and CD80 were not expressed in the testes of the transgenic mice, and therefore, the PD‐1/PD‐L1 and PD‐L1/CD80 pathways were not activated. Receptor‐ligand interactions are at the base of all biological events occurring in living cells. This suggested that PD‐L1 is not just a ligand and possibly a receptor for self‐activation. Such molecules with self‐activating effects are rarely reported. Recently, a self‐activating GPCR transmembrane protein was reported, and its activity is not activated by ligand molecules but relies exclusively on its own particular mechanism, the built‐in activated state conformation formed by the ECL2 domain.^44^ Escors et al. found that the intracellular fragment of PD‐L1 contains a signal transduction domain that plays a pro‐proliferative and pro‐apoptotic role in interferons.^20^ This suggested that it is highly likely that PD‐L1, such as GPCRs, acts both as a receptor and as a ligand, self‐activating and then signalling intracellularly. The present study found that germ cell sloughing occurs in the testes of PD‐L1 transgenic mice in the absence of ligand activation. Further study found that PD‐L1 could bind with each other and influence the expression of related adhesion factors.

PD‐L1 is highly expressed in various malignancies, including non‐small cell lung cancer, melanoma, renal cell carcinoma, prostate cancer, breast cancer and glioma.^45^ PD‐L1 has been reported to be able to enhance the metastatic ability of tumours, leading to increased patient mortality.^46^ Thompson et al. found that PD‐L1 expression was significantly associated with tumour metastasis and death in patients.^47^ Zhang et al. found that PD‐L1 expression in lung adenocarcinoma was associated with T stage (T2‐T4) and N stage (N1/N2) of progression.^48^ Muenst et al. found that high expression of PD‐L1 was negatively associated with prognosis in breast cancer patients.^49^ In 2008, PD‐L1 was first reported as a molecular barrier to tumour protection independent of PD‐1 for signal transduction. Only when tumour cells express PD‐L1 can they defend against attack by T lymphocytes, but this process is independent of whether T lymphocytes express PD‐1. In another study, ectopic expression of PD‐L1 on colon cancer cell lines in vitro found that PD‐L1, in addition to binding to PD‐L1, promoted the proliferation and migration and downregulated the adhesion of colon cancer cells through the EMT pathway (submitted). These results suggest a link between the role of PD‐L1 in promoting EMT and its ability to mediate tumour cell adhesion, but the specific mechanism requires further investigation. Therefore, the significance of this study also reveals that PD‐L1 may promote tumour development and metastasis through self‐interaction in tumour research.

It is believed that infections of the male reproductive tract contribute substantially to impaired fertility. Up to 15% of male fertility disorders can be attributed to infections and inflammatory conditions.^50^ In the male reproductive system, although the testis is an immune organ, infections and inflammation (e.g., orchitis, bacterial epididymitis and sterile orchitis) may disrupt immunosuppressive mechanisms and induce autoimmune reactions against sperm antigens, resulting in azoospermia and infertility. Quantitative and phenotypic analysis of testis‐infiltrating cells showed an increase in immune cells and secretion of the proinflammatory cytokines TNF‐α, IFN‐γ, IL‐6, IL‐12, IL‐17 and IL‐23, which also disrupts the normal testicular immunosuppressive microenvironment.^51^ Studies have shown that both histiocytes (e.g., macrophages and epithelial cells) and tumour cells promote PD‐L1 expression through the activation of JAK and STAT3 in response to proinflammatory factors.^52^, ^53^ This suggested to us that testicular inflammation might activate the related signalling pathway through PD‐L1/PD‐L1 self‐activation, thus causing the shedding of spermatogonia and finally male infertility. Combined with our findings, the specific mechanism of this hypothesis needs to be confirmed by further studies. In addition, it is reported that peritubular myoid cells can form a basement membrane in seminiferous tubules with Sertoli cells to provide a niche for spermatogonial stem cells to self renew.^54^ Peritubular myoid cells at the periphery of seminiferous tubules prevents direct physical contact between Sertoli cells and Leydig cells. Leydig cells, present in the interstitial space between the seminiferous tubules, are the primary source of androgens. Abnormalities in interstitial cells versus peritubular cells cause altered tube wall structure, which leads to the altered function. For example, it is well‐known that testicular tubular fibrosis, best evidenced by deposits of ECM, is considered a hallmark of male infertility. Fibrosis and morphological changes of peritubular cells may imply that specific functions of peritubular cells such as paracrine are altered. At present, there are no more reports to study the effect of PD‐L1 on peritubular cells and Leydig cell, which also needs our further research in the future.

In summary, this study identifies a role for PD‐L1 in causing abnormal sperm development and detachment of germ cells. We found that PD‐L1 overexpression by Sertoli cells and spermatids causes a failure in the later stages of spermatogenesis characterized by abnormal sperm morphology and function, ultimately leading to male infertility. PD‐L1 can interact with PD‐L1, suggesting its regulatory role in microtubule organization and cell adhesion function, which needs to be verified in future studies. The effect of PD‐L1 on germ cells may indicate a universal effect of PD‐L1 on cell adhesion function.

## CONFLICT OF INTEREST

The authors declare no conflict of interest.

## AUTHOR CONTRIBUTIONS

**Lian Fang:** Conceptualization (equal); Methodology (equal); Writing – original draft (equal). **Rui Feng:** Conceptualization (equal); Methodology (equal); Writing – original draft (equal). **Weiye Liang:** Conceptualization (equal); Methodology (equal); Writing – review & editing (equal). **Fangfang Liu:** Conceptualization (equal); Methodology (equal); Writing – original draft (equal). **Ganlan Bian:** Formal analysis (lead); Visualization (equal). **Caiyong Yu:** Investigation (equal); Validation (equal). **Hongmin Guo:** Validation (equal); Visualization (equal). **Yihui Cao:** Investigation (equal); Visualization (equal). **Mingkai Liu:** Formal analysis (equal); Visualization (equal). **Jia Zuo:** Formal analysis (equal); Visualization (equal). **Yinglong Peng:** Formal analysis (equal); Visualization (equal). **Jie Zhao:** Investigation (equal); Validation (equal). **Rui‐Xia Sun:** Resources (equal); Supervision (equal); Writing – original draft (equal). **Jiajie Shan:** Resources (equal); Supervision (equal); Writing – original draft (equal). **Jian Wang:** Funding acquisition (lead); Resources (lead); Writing – review & editing (equal).

## Supporting information

Fig S1

Fig S2

Fig S3

Table S1

## Data Availability

Data sharing is not applicable.
