# Supplementary figures and images for: Overexpression of PD‐L1 causes germ cells to slough from mouse seminiferous tubules via the PD‐L1/PD‐L1 interaction
Source: J Cell Mol Med. 2022 Apr 5;26(10):2908–20. doi: 10.1111/jcmm.17305 (PMC9097848; doi:10.1111/jcmm.17305)

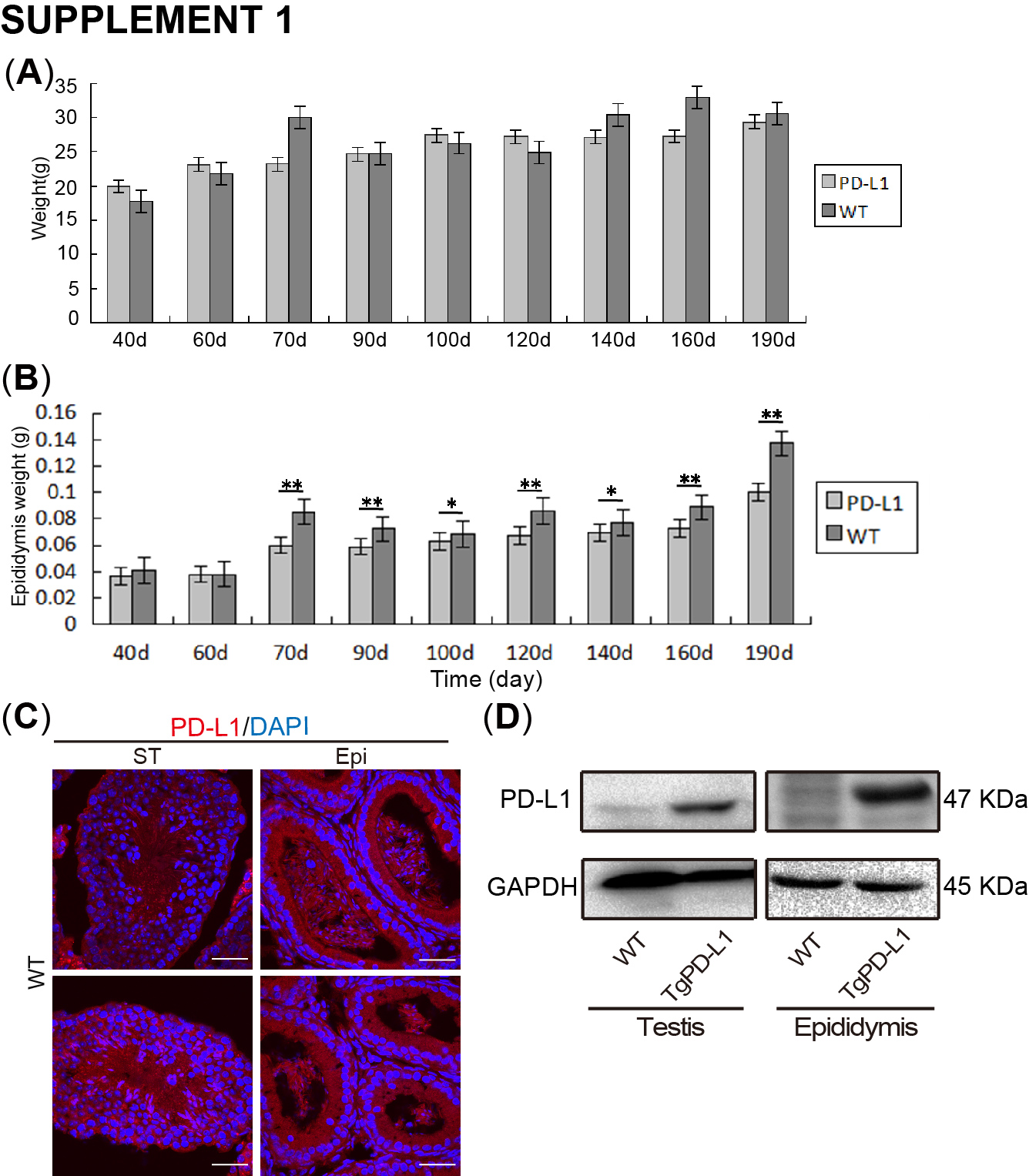

Supplement: Supplementary file 1 — Fig S1 [file JCMM-26-2908-s004.jpg]

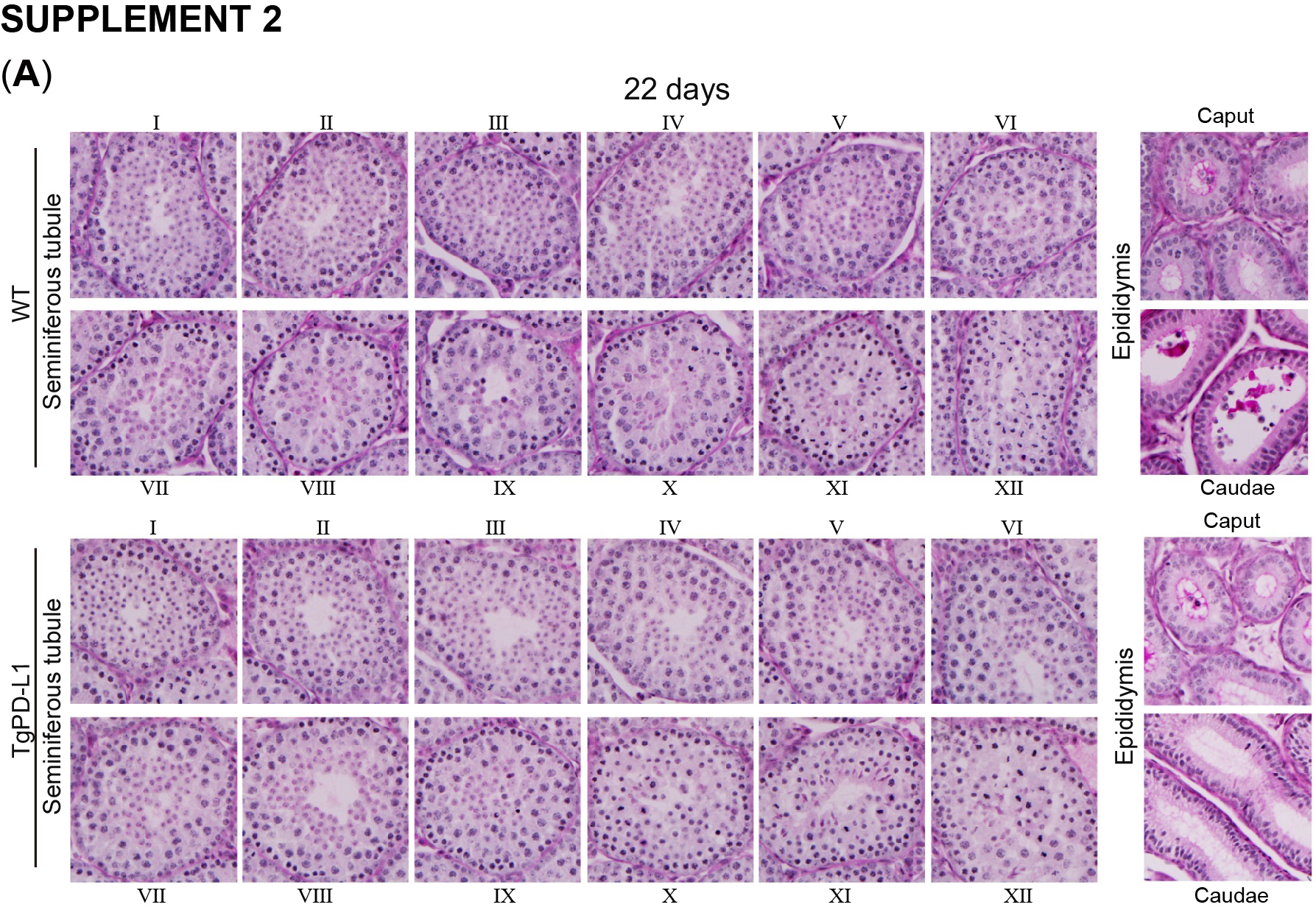

Supplement: Supplementary file 2 — Fig S2 [file JCMM-26-2908-s003.jpg]

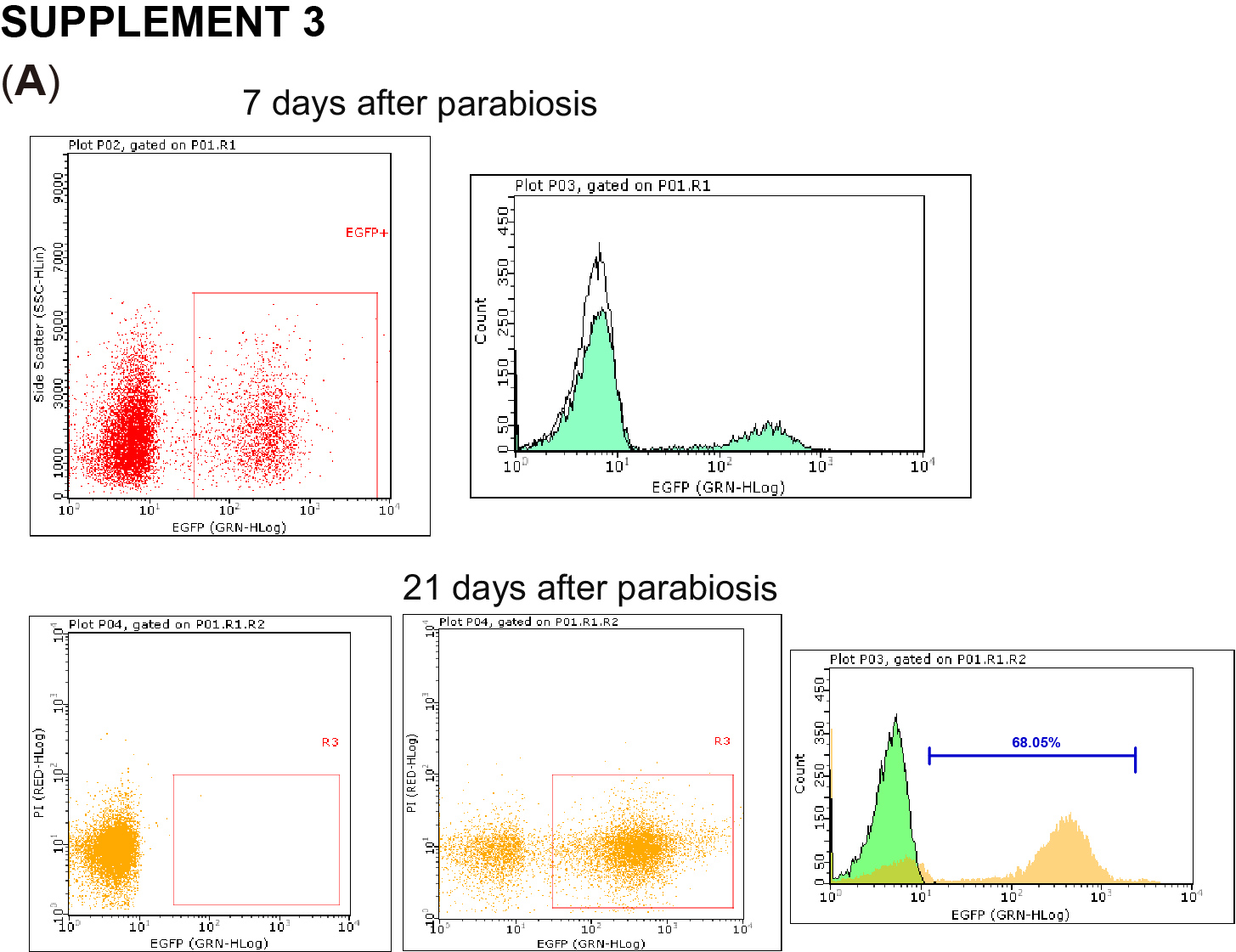

Supplement: Supplementary file 3 — Fig S3 [file JCMM-26-2908-s001.jpg]
