# Supplementary material for: Overexpression of PD‐L1 causes germ cells to slough from mouse seminiferous tubules via the PD‐L1/PD‐L1 interaction
Source: J Cell Mol Med. 2022 Apr 5;26(10):2908–20. doi: 10.1111/jcmm.17305 (PMC9097848; doi:10.1111/jcmm.17305)
Supplement: Supplementary file 4 — Table S1 [file JCMM-26-2908-s002.docx]

Supplementary Table 1. Primer sets for qRT-PCR

| For qRT-PCR | | | |
| --- | --- | --- | --- |
| Gene | Species | Forward (5’-3’) | Reverse (5’-3’) |
| GAPDH | Mouse | TGAAGCAGGCATCTGAGGG | CGAAGGTGGAAGAGTGGGAG |
| PD-L1 | Mouse | GAGAGCCACGTCACACTGG | CAAAGTCGGCGTAGTCAAGC |
| CD80 | Mouse | ACCCCCAACATAACTGAGTCT | TTCCAACCAAGAGAAGCGAGG |
| PD-1 | Mouse | CAGCTTGTCCAACTGGTCG | GCTCAAACCATTACAGAAGGCG |
| CTLA-4 | Mouse | CATGGTGTCGCCAGCTTTC | GGTAATCTAGGAAGCCCACTGTA |
| GAPDH | Human | TCCAAAATCAAGTGGGGCGAT | TTCTAGACGGCAGGTCAGGTC |
| PD-L1 | Human | TGGCATTTGCTGAACGCATTT | TGCAGCCAGGTCTAATTGTTTT |
| For PCR | | | |
| Gene | Species | Forward (5’-3’) | Reverse (5’-3’) |
| PD-L1 | Mouse | GAGGATATTTGCTGGCATTATATTAT | TTACGTCTCCTCGAATTGTGTATCA |
